# Prognostic impact of lymphocyte kinetics and immune composition during bispecific antibody therapy in relapsed/refractory multiple myeloma

**DOI:** 10.1038/s41408-026-01613-9

**Published:** 2026-08-22

**Authors:** Mansi R. Shah, Aishee Bag, Asis Shrestha, Yetunde Ogunsesan, Anup Trikannad, Sruthi Vellanki, Sanjay Muttineni, Hira Cheema, Syed Naqvi, Sasya Dronavalli, Samer Al Hadidi, Sharmilan Thanendrarjan, Maurizio Zangari, Frits van Rhee, Aniko Szabo, Rajshekar Chakraborty, Meera Mohan, Carolina Schinke

**Affiliations:** 1 Division of Blood Disorders, Rutgers Cancer Institute, New Brunswick, NJ USA; 2 Department of Internal Medicine, Rutgers Robert Wood Johnson Medical School, New Brunswick, NJ USA; 3 University of Arkansas for Medical Sciences, Myeloma Center, Little Rock, AR USA; 4 Division of Hematology Oncology, Medical College of Wisconsin, Milwaukee, WI USA; 5 Myeloma, Waldenstrom’s, and Amyloidosis Program, Simmons Comprehensive Cancer Center, UT Southwestern Medical Center, Dallas, TX USA; 6 Division of Biostatistics, Data Science Institute, Medical College of Wisconsin, Milwaukee, WI USA; 7 Division of Division of Hematology Oncology, Columbia University Irving Medical Center, New York, NY USA; 8 Division of Lymphoma and Myeloma, University of Texas MD Anderson Cancer, Houston, TX USA

**Keywords:** Translational research, Haematological cancer

Letter to the editor

Bispecific antibodies (bsAbs) targeting B-cell maturation antigen (BCMA) and G protein–coupled receptor class C group 5 member D (GPRC5D) have become important therapeutic options for patients with relapsed/refractory multiple myeloma (RRMM), producing overall response rates of up to 70% [1–4]. Despite these advances, clinical outcomes remain heterogeneous, and because bsAbs rely on sustained engagement of immune effector cells, treatment efficacy is dependent on the fitness of the immune compartment. As a result, readily available immune biomarkers, such as absolute lymphocyte count (ALC) and lymphocyte subset composition-, including CD4 and CD8 T cells, B cells, and natural killer (NK) cells—may capture qualitative differences in immune competence that influence the likelihood of response to bsAb therapy [5]. While studies of CAR-T therapies in RRMM have shown that baseline lymphopenia and impaired lymphocyte expansion correlate with adverse clinical outcomes [6, 7], the prognostic impact of lymphocyte dynamics during bsAb therapy remains unclear.

To evaluate the impact of baseline ALC, early ALC kinetics, and lymphocyte subset trajectories, we conducted a retrospective, multi-institutional analysis of RRMM patients treated with at least one full dose of BCMA-or GPRC5D-directed bsAb. Patients were treated across four academic centers in the United States: University of Arkansas for Medical Sciences, Rutgers Cancer Institute, Columbia University Irving Medical Center, and Medical College of Wisconsin. Patient and disease characteristics were collected prior to the start of therapy. High-risk cytogenetics were defined as the presence of translocations t(4;14); t(14;16); t(14;20); gain/amplification of 1q21, del(1p), or del(17p) by FISH. ALC data were available in 303 patients and were collected at baseline and at prespecified on-treatment time points (days 0 [first step-up dose (SUD)], 3, 7, 14, 30, 90, and 180). Lymphocyte subsets were available in 124 patients and were collected on days 0, 30, 90, and 180. Lymphocyte antibody panel included CD3, CD45, CD16, CD56, CD4, CD8, and CD19. NK cells were defined as CD3-negative, CD16-positive, and CD56-positive. Lymphopenia was defined as ALC < 1.0 × 10³/µL. Response was assessed according to the International Myeloma Working Group (IMWG) criteria, and “responders” were defined as having achieved at least a partial response (PR). All statistical analyses can be found in the supplemental material.

The median age at treatment initiation was 70 years, and 159 patients (52%) were male (Table 1). BCMA-directed bsAbs were administered in 217 (72%) patients, and 86 (28%) received GPRC5D-targeted bsAbs. The median number of prior lines of therapy was 5 (range, 1–14). High-risk cytogenetics were seen in 160 (53%) of patients, the median baseline ALC was 0.91 × 10^3^/ µL, and lymphopenia was present in 158 patients (52%). Del(17p) and del(1p) were more frequent among non-responders (*p* < 0.05), and gain/amplification of 1q21 also trended toward association with inferior response (*p* = 0.056). Response rates differed by prior autologous stem cell transplant (ASCT) status, with more responders having no prior ASCT (*p* = 0.03). Responders also had significantly higher baseline hemoglobin and platelet counts (*p* < 0.01). In contrast, baseline lymphopenia was not associated with response, and baseline ALC was similar between responders and non-responders (0.91 vs. 0.92 K/µL).

**Table 1:** Baseline characteristics stratified by objective response.

Characteristic	Overall (*N* = 303)	Responders (*N* = 215)	Non-Responders (*N* = 88)	*p*-value
Age, median (range)	70 (33 to 91)	70 (33 to 90)	70 (43–91)	0.77
Sex, *n* (%)				0.84
Male	159 (52%)	112 (52%)	47 (53%)	
Female	144 (48%)	103 (48%)	41 (47%)	
Race/Ethnicity, *n* (%)				0.35
Non-Hispanic White	210 (69%)	154 (72%)	56 (64%)	
Non-Hispanic Black	64 (21%)	40 (19%)	24 (27%)	
Hispanic/Other	29 (10%)	21 (9%)	8 (9%)	
Target, *n* (%)				0.78
BCMA	217 (72%)	153 (71%)	64 (73%)	
GPRC5D	86 (28%)	62 (29%)	24 (27%)	
High-Risk Cytogenetics, *n* (%)	160 (53%)	104 (48%)	56 (64%)	0.051
translocation t4;14	30 (10%)	18 (8%)	12 (14%)	0.25
translocation t14;16	14 (4.6%)	11 (5%)	3 (3.3%)	0.56
del(17p)	62 (20%)	35 (16%)	27 (31%)	**0.009**
del(1p)	47 (18%)	26 (12%)	21 (24%)	**0.038**
1q21 gain	100 (40%)	62 (29%)	38 (43%)	0.056
Prior Lines of Therapy, median (range)	5 (1–14)	5 (2–14)	5 (1–12)	0.59
Prior ASCT, *n* (%)				**0.03**
0	58 (19%)	48 (22%)	10 (11%)	
1	161 (53%)	102 (47%)	59 (67%)	
≥2	84 (28%)	60 (28%)	24 (27%)	
Triple-Class Refractory	266 (88%)	193 (90%)	73 (83%)	0.11
Hemoglobin (g/dL), median (range)	10.1 (5–17)	10.4 (6–16.5)	9.6 (5–17)	**0.003**
Platelets (K/µL), median	134 (5–424)	140 (5–424)	104 (7–392)	**0.008**
Absolute Lymphocyte count (x10^3^/ µL), range	0.91 (0–6.50)	0.9 (0–6.50)	0.91 (0.05–4.47)	0.25
Baseline Lymphopenia(<1.0 K/µL)	158 (52%)	112 (52%)	46 (52%)	0.98
Baseline ALC, median (K/µL)	0.91 (0–650)	0.91 (0.05–4.47)	0.92 (0.9–6.5)	0.31

*ASCT* autologous stem cell transplant, *bsAb* bispecific antibody, *HR* high-risk, *IMWG* International Myeloma Working Group, *ORR* overall response rate.

Baseline lymphopenia was not associated with progression-free survival (PFS), with similar 2-year PFS rates of 23% and 28% (Supplementary Fig. 1A). There was also no significant difference in overall survival (OS) by baseline lymphopenia status, although 2-year OS was numerically higher among patients without lymphopenia, 55% vs 42% (Supplementary Fig. 1B**)**.

We then evaluated ALC dynamics over time and observed a significant decline during the first week after bsAb initiation, decreasing from a median baseline of 1100/μL to 300/μL at day 7, (*p* < 0.001), followed by recovery to levels comparable to baseline by day 90 (Fig. 1A**)**. This pattern was observed in both responders and non-responders; however, responders demonstrated a significantly faster expansion with higher ALC levels at Day 14 (*p* = 0.001; Fig. 1B**)**.

**Fig. 1:**
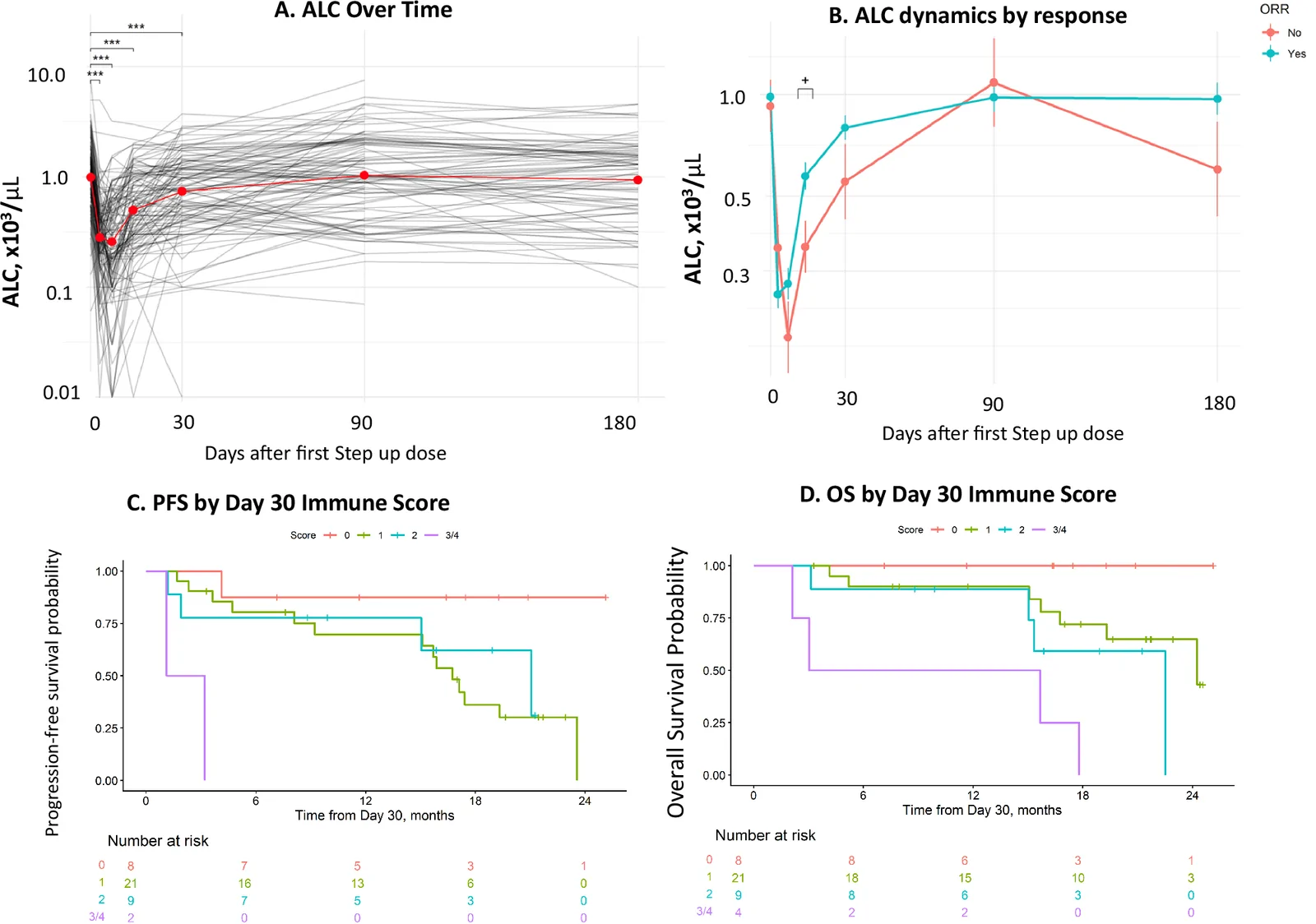
Lymphocyte kinetics and exploratory immune subset score during bispecific antibody therapy. **A** Absolute lymphocyte count (ALC) over time in the overall cohort. **B** ALC over time stratified by objective response. **C** Progression-free survival by day 30 immune score. **D** Overall survival by day 30 immune score. Responders were defined as patients achieving at least partial response by International Myeloma Working Group criteria. + *p* = 0.001, ***p < 0.001.

We next investigated the dynamics of lymphocyte subsets. CD4 T-cell counts decreased from a median of 283/μL at baseline to 216/μL at Day 30 (*p* = 0.048), with recovery toward baseline thereafter (Supplementary Fig. 2A). This decrease appeared to be driven mainly by non-responders who had substantially lower CD4 T cell counts at D 30, *p* = 0.029 (Supplementary Fig. 2B). CD19 B-cell counts showed a rapid and sustained decline over time, decreasing from a median baseline of 32/μL to approximately 20/μL or lower at subsequent time points (days 30, 90 and 180), *p* < 0.001 (Supplemental Fig. 2C) with no significant difference between respondersn and non-responders (Supplemental Fig. 2D). In contrast, CD56 NK-cell counts increased steadily over time, rising from 110/μL at baseline to 155/μL by day 180, *p* < 0.001, with substantially higher CD56 counts being observed among responders, albeit this was not significant (Supplementary Fig. 2E-F**)**. CD8 T-cell counts declined at day 30 (median 528/μL to 432/μL; *p* = 0.031), with subsequent recovery to baseline values (Supplemental Fig. 2G-H**)**.

Lastly, in exploratory analyses among patients with available lymphocyte subset data (*n* = 40), CD4/CD8 ratio, CD19 B-cell counts, and CD56 NK-cell counts at Day 30 showed the strongest associations with PFS and OS. Based on these observations, we constructed an exploratory scoring model incorporating these 30-day variables: CD4/CD8 ratio < 0.5 (1 point), CD19 > 20/µL (2 points), and CD56 < 135/µL (1 point). When patients were stratified by cumulative score, higher scores were associated with shorter post-D30 PFS. Median PFS from D30 was 1 month for those with a score of 3/4, whereas median PFS was not reached among patients with a score of 0, *p* < 0.001. (Fig. 1C**)**. Similarly, median post-D30 OS was shorter among patients with a score of 3/4 compared with those with a score of 0 (3 months vs. not reached, *p* < 0.001; Fig. 1D). The D30 score was predictive of OS (HR = 3.0, 95% CI: 1.6–5.4, *p* < 0.001) and non-significantly associated with PFS (HR = 2.0, 95% CI: 0.9 – 4.3, *p* = 0.09) when adjusted for baseline age, high risk disease, number of prior ASCT, presence of PD at bsAb initiation, hemoglobin, serum albumin and thrombocytopenia through propensity-based weighting.

Taken together, this study did not find a significant association of baseline lymphocyte count and response. Similarly, a previous study showed that baseline ALC of ≤0.5 × 10³/μL had no adverse prognostic impact [8], suggesting that pretreatment lymphocyte counts alone may have limited utility for identifying patients most likely to derive benefit from bsAb therapy.

In contrast, longitudinal immune dynamics during therapy differed between response groups—with a more rapid and profound expansion and early ALC recovery amongst responders. Intriguingly, Banerjee et al. similarly showed that ALC expansion at D + 30 is associated with delayed response, while early ALC counts did not differ between responders and non-responders [9]. These findings support the concept that early immune effector recovery parallels observations in CAR-T cell therapy, where more robust lymphocyte expansion has been associated with improved clinical outcomes [6].

We also observed differential patterns in lymphocyte subsets over time, although data were available at limited time points and early changes during SUD may not have been fully captured. A higher CD4 T-cell count was observed in responders compared with non-responders, suggesting that CD4 T-cell recovery may be relevant to response during bsAb therapy. Intriguingly, in CAR-T cell therapy, higher CD4 T cell levels in the apheresis products have been associated with better efficacy and sustained CAR-T persistence [10]. In our analyses, a low CD4/CD8 T-cell ratio was incorporated into a simplified immune scoring model and was associated with inferior survival. These findings raise the possibility that CD4 T-cell fitness and recovery may represent an important marker of bsAb efficacy.

Additionally, CD19 B-cell counts demonstrated a sustained decline over time. The biologic interpretation of peripheral CD19 dynamics in this setting remains uncertain. CD19 has been implicated in less differentiated myeloma stem cell populations which are enriched for tumor-propagation and drug resistance. Consequently, eradication of the CD19-positive subpopulation has been hypothesized to improve outcomes, and CD19/BCMA dual-targeting CAR-T cell strategies are currently under investigation [11].

Finally, the role of CD56-positive Natural Killer (NK)/T-cells in T-cell redirection therapy has not been well described, but impaired activity is suggestive of adverse outcomes [12]. In our cohort, CD56 positive counts increased over time, and higher CD56 counts were associated with better prognosis in our exploratory scoring model. These findings suggest that broader immune remodeling beyond T-cell subsets may be relevant during bsAb treatment and warrants further mechanistic investigation.

Importantly, the observed immune alterations are not only clinically relevant but potentially targetable. Specifically, immunomodulatory drugs (IMiDs) and Cereblon E3 Ligase Modulatory Drugs (CELmoDs) have been shown to enhance immune cells, including CD4 T-cells and CD56 NK-cell activity, and may provide rational partners for bsAb combinations to overcome immune factors associated with poor response [13–15]. Broadly, our findings suggest that on-treatment lymphocyte kinetics and immune composition may provide prognostic information beyond baseline ALC alone and identify patients at risk for poor outcomes. Our exploratory scoring model should be viewed as hypothesis-generating and mainly serves to reflect immune alterations that are associated with prognosis and could play an important biological role to optimize efficacy of immunotherapy. Until these immune parameters are validated prospectively, overall clinical assessment should remain the primary basis for treatment decisions.

## Supplementary information

Supplementary Material

## Data Availability

Data may be requested from the corresponding study authors.
