# Supplementary material for: Prognostic impact of lymphocyte kinetics and immune composition during bispecific antibody therapy in relapsed/refractory multiple myeloma
Source: Blood Cancer J. 2026 Aug 22;16(1):141. doi: 10.1038/s41408-026-01613-9 (PMC13499802; doi:10.1038/s41408-026-01613-9)
Supplement: Supplementary file 1 [file 41408_2026_1613_MOESM1_ESM.docx]

Supplemental Material

## Statistical Methods

Continuous variables, including baseline ALC, were compared between responders and non-responders using the Wilcoxon rank-sum test. Categorical variables, including baseline lymphopenia, were compared using Pearson’s χ² test or Fisher’s exact test. Fold-changes from baseline and by ORR status with corresponding 95% confidence intervals (CIs) were estimated using log-transformed linear mixed effect models. All statistical analyses were performed using R version 4.2.0.

## *Time-to-event analyses*

Overall survival (OS) was defined as the interval from initiation of bispecific antibody therapy to death from any cause or last follow-up. Progression-free survival (PFS) was defined as the interval from treatment initiation to disease progression or death, whichever occurred first. Survival distributions were estimated using the Kaplan–Meier method, with survival probabilities reported at 12 and 24 months together with median survival estimates. Survival curves were compared according to baseline lymphopenia status using log-rank tests.

## *Modeling of ALC and lymphocyte subset dynamics*

For repeated-measures analyses, zero values were replaced with one-half of the smallest positive observed value within each marker and measurements were analyzed on the log scale. Linear mixed-effects models with a random patient intercept and fixed day effect were fitted to evaluate temporal changes in lymphocyte subsets. Estimated marginal means were used to calculate fold-changes relative to baseline, and multiplicity-adjusted pairwise comparisons of each post-baseline time point versus baseline were generated using multivariate t-distribution based adjustment. Overall tests were obtained as joint F tests of the same contrasts.

To assess associations between lymphocyte trajectories and treatment response, mixed-effects models including fixed effects for day, ORR status, and their interaction were fitted. Estimated marginal means were used to compare responders and non-responders at each time point, with adjustment for multiple comparisons. Overall tests were obtained as joint F tests of the same contrasts.

## *Effect of lymphocyte subset dynamics on OS and PFS*

To evaluate prognostic effects of early lymphocyte recovery, 30-day landmark analyses were performed. Patients were required to remain alive and under follow-up for OS analyses and free of progression for PFS analyses through day 30. Baseline and early lymphocyte subset measurements (days 0, 3, 7, 14, and 30) were log2-transformed after replacement of zero values with one-half the smallest positive observed value. Hazard ratios therefore represent the effect of a two-fold increase in the biomarker value.

Univariable Cox proportional hazards models were fitted for each lymphocyte measurement. Multivariable Cox models incorporating multiple lymphocyte variables were then fitted, and Akaike information criterion (AIC)-based backward model selection was used to identify parsimonious prognostic models. Separate models were developed for OS and PFS.

## *Development of a simplified prognostic score*

Based on the final landmark models, a simplified risk score was developed using day-30 lymphocyte subset measurements. CD4 and CD8 effects were combined as the CD4/CD8 ratio because their regression coefficients demonstrated similar magnitudes and opposite directions. Biomarkers were dichotomized using median-based cutoffs: CD4/CD8 ratio <0.5, CD19 >20 cells/µL, and CD56 <135 cells/µL. Integer point values were assigned according to the magnitude of model coefficients, yielding a score ranging from 0 to 4. Model performance was evaluated using likelihood ratio statistics, AIC, and concordance indices. Kaplan–Meier curves were generated according to score category.

## *Propensity-weighted analyses*

To evaluate the independent prognostic impact of the simplified score, inverse probability weighting was performed using baseline clinical covariates, including age, high-risk cytogenetics, number of prior autologous stem cell transplants, disease status at bispecific antibody initiation, hemoglobin, serum albumin, and thrombocytopenia. Weighted Cox proportional hazards models were then fitted for both OS and PFS.

## Subject selection for analyses

|  | Cohort | Number of patients included | Number of patients excluded |
| --- | --- | --- | --- |
| 1. | Study dataset | 303 |  |
| 2. | OS analysis | 300 | 3 – unknown survival status |
| 3. | PFS analysis | 294 | 6 – unknown progression status |
| 4. | Lymphocyte subset kinetics analyses | 124 | 170 – data not collected |
| 5. | Lymphocyte subset association with response | 118 | 6 – non-evaluable or unknown response |
| 6. | OS landmarked at D30 | 114 | 4 – died or lost to follow-up before D30 |
| 7. | PFS landmarked at D30 | 106 | 8 – progressed before D30 |
| 8. | OS D30 immune score model | 42 | 72 – not all required subset counts available (vs Cohort 6) |
| 9. | PFS D30 immune score model | 40 | 66 – not all required subset counts available (vs Cohort 7) |

**Supplemental Figure 1**. Survival outcomes by baseline lymphopenia status. (A) Progression-free survival stratified by baseline lymphopenia. (B) Overall survival stratified by baseline lymphopenia. Lymphopenia was defined as ALC <1.0 ×10³/µL at baseline.

**Supplemental Figure 2.** Lymphocyte subset trajectories during bispecific antibody therapy. Changes in lymphocyte subset counts over time in the overall cohort and stratified by response status, including CD4 T cells, CD19 B cells, CD56 NK cells, and CD8 T cells.

H

G

F

E

D

C

B

A
